# Recent physical connections may explain weak genetic structure in western Alaskan chum salmon (*Oncorhynchus keta*) populations

**DOI:** 10.1002/ece3.628

**Published:** 2013-06-13

**Authors:** Michael R Garvin, Christine M Kondzela, Patrick C Martin, Bruce Finney, Jeffrey Guyon, William D Templin, Nick DeCovich, Sara Gilk-Baumer, Anthony J Gharrett

**Affiliations:** 1 School of Fisheries and Ocean Sciences, University of Alaska Fairbanks17101 Point Lena Loop Road, Juneau, Alaska, 99801; 2 Auke Bay Laboratories, Alaska Fisheries Science Center, National Oceanic and Atmospheric Administration, Ted Stevens Marine Research Institute17109 Point Lena Loop Road, Juneau, Alaska, 99801; 3 Concerned Area M Fishermen, 2771 Deer Creek DriveBozeman, Montana, 59715; 4 Departments of Biological Sciences and Geosciences, Idaho State UniversityPocatello, Idaho, 83209-8007; 5 Alaska Department of Fish and Game, Division of Commercial Fisheries333 Raspberry Road, Anchorage, Alaska, 99518

**Keywords:** Chum salmon, isolation-by-distance, landscape genetics, microsatellite, population genetics, SNP

## Abstract

Low genetic divergence at neutral loci among populations is often the result of high levels of contemporary gene flow. Western Alaskan summer-run chum salmon (*Oncorhynchus keta*) populations demonstrate weak genetic structure, but invoking contemporary gene flow as the basis for the low divergence is problematic because salmon home to their natal streams and some of the populations are thousands of kilometers apart. We used genotypes from microsatellite and single nucleotide polymorphism loci to investigate alternative explanations for the current genetic structure of chum salmon populations from western Alaska. We also estimated current levels of gene flow among Kuskokwim River populations. Our results suggest that weak genetic structure is best explained by physical connections that occurred after the Holocene Thermal Maximum among the Yukon, Kuskokwim, and Nushagak drainages that allowed gene flow to occur among now distant populations.

## Introduction

The genetic structure of populations results from contemporary and historical processes. Typically, allele frequencies at many loci explain the divergence among sub-populations and a vicariant or a dispersal event associated with their geography is used to explain the reason for that divergence (see Avis 2000). The genetic divergence at neutral loci often stems from genetic drift, and the strength of the divergence is directly proportional to the number of generations since the vicariant or dispersal event, but inversely proportional to the size and gene flow among sub-populations. Gene flow may have been continual or may have occurred following generations of separation. Conversely, genetic structure among populations can result from selective processes; large divergences may exist at loci if they provide adaptation to local environments, but small divergences could be displayed at loci under purifying selection.

**Figure 1:**
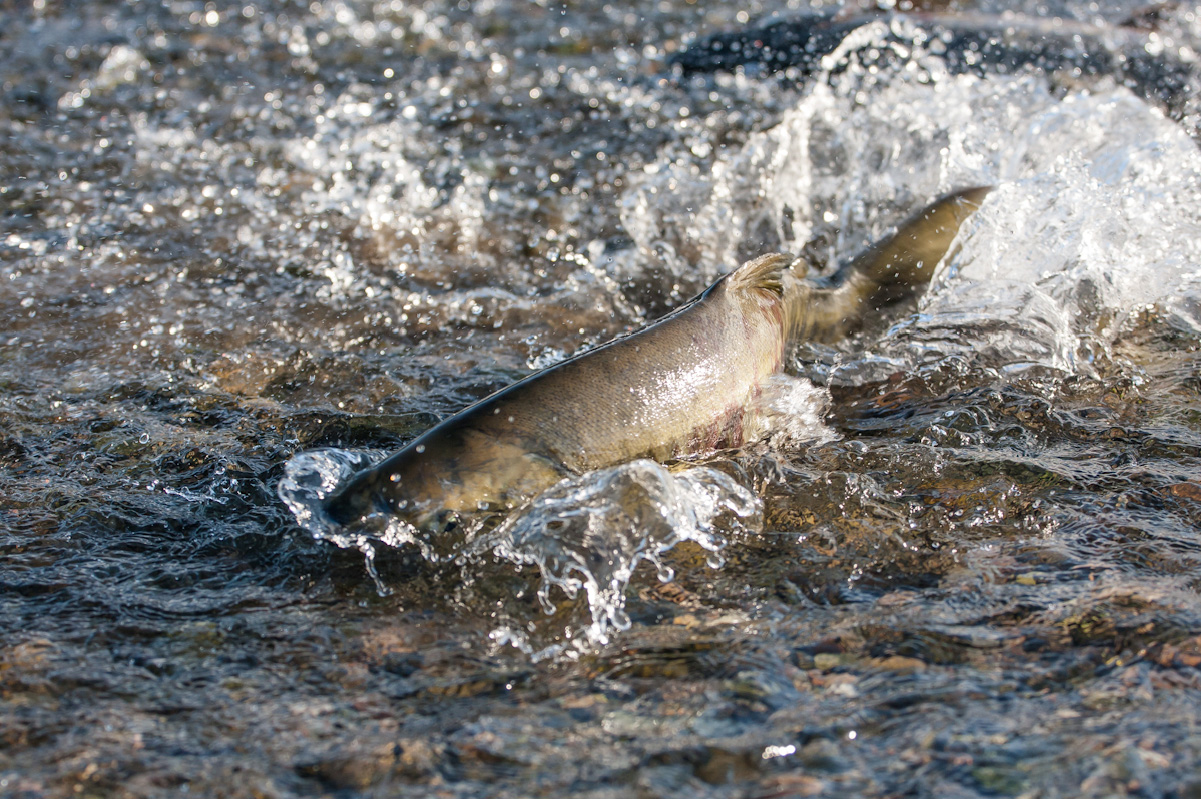
An adult chum salmon (*Oncorhynchus keta*) ascending a stream to spawn (photo with permission from Patrick Barry).

**Figure 2:**
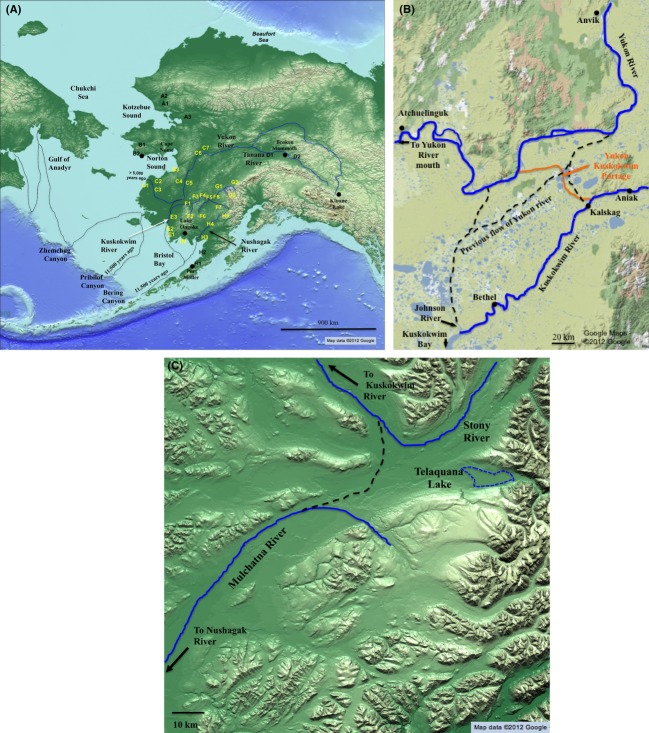
(A) Area of study and populations (identified with alpha-numeric codes; Table 1). Coastal southwestern Alaska (CSW) are in yellow. Current drainages are indicated with blue lines and black broken lines indicate known and likely historical river systems taken from Creager and McManus (1967) and Shepard and Wanless (1971). (B) Historical Yukon-Kuskokwim river connections at two locations (black broken lines) and the current Yukon-Kuskokwim Portage (orange line) taken from Shepard and Wanless (1971). The villages of Anvik, Bethel, Kalskag, Atchuelinguk, and Aniak provide reference points. (C) Historical Kuskokwim-Nushagak river connections between the upper Nushagak (Mulchatna) River and the Middle Kuskokwim (Stony) River. Current river systems are indicated with blue lines and the likely historical river connection taken from Maddren (1910) is indicated with a black broken line. The location of Telaquana Lake is outlined with blue broken lines.

Chum salmon (*Oncorhynchus keta,* Figure 1) typically have lower levels of divergence compared to other Pacific salmon species such as Chinook (*Oncorhynchus tshawytscha*), sockeye (*Oncorhynchus nerka*), coho (*Oncorhynchus kisutch*), and steelhead (*Oncorhynchus mykiss*); Quinn 2005; Olsen et al. 2010; Seeb et al. 2011; Templin et al. 2011), which may be because chum salmon have larger effective population sizes and do not have a freshwater phase that might increase divergence among populations. The populations from the large geographic area that includes Kotzebue Sound, Norton Sound, the Lower Yukon River, the Kuskokwim River, and Bristol Bay (Fig. 2A) demonstrate much lower genetic divergence than populations from the remainder of the chum salmon range (Seeb and Crane 1999; Beacham et al. 2009a; Seeb et al. 2011). Low divergence has been attributed to recent dispersal into western Alaska following the last glacial maximum (LGM) (Wilmot et al. 1994). A subset of summer-run chum salmon populations nested within western Alaska that we call “coastal southwestern Alaska” (CSW) span a geographic area of approximately 350,000 km^2^ (roughly the size of Washington, Oregon, and Idaho combined) and yet they display genetic divergence that is 5–10 times lower than the rest of western Alaska (Seeb and Crane 1999; Beacham et al. 2009b; Seeb et al. 2011).

The weak genetic structure of CSW populations could be from several causes. Positive diversifying selection may have produced the genetic similarity among CSW populations, but this is unlikely because the large geographic region includes several different ecosystems (Olsen et al. 2010); one would not expect similar allele frequencies to be maintained at loci responsible for local adaptation among populations from different environmental regimes. Moreover, only a few loci would likely be affected, but numerous loci indicate weak structure (Seeb et al. 2004, 2011; Beacham et al. 2009b). It has been suggested that contemporary gene flow maintains the reduced divergence among CSW populations (Utter et al. 2009; Olsen et al. 2010), but the smallest divergence estimates are between populations that are thousands of kilometers apart. Divergent populations of Pacific salmon often demonstrate an isolation-by-distance (IBD) pattern, which is consistent with the concept that populations in close proximity show less divergence than more distant ones because proximal populations exchange more migrants. In the case here, weak genetic structure among geographically distant populations would result only if substantial gene flow occurred serially between neighboring populations for many generations or if long distance dispersals were common.

One untested hypothesis is that historical physical connections among CSW drainages allowed gene flow to erode genetic divergence among CSW populations. Some of these physical connections could have reduced corridors among populations from thousands of kilometers to tens of kilometers. Geological data indicate that connections occurred between the Lower Yukon and Kuskokwim rivers in at least two locations (Creager and McManus 1967; Shepard and Wanless 1971). The first was near the village of Kalskag at the existing Yukon-Kuskokwim portage (Dougan 2010); and the second was through what is now the Johnson River, which empties into the lower Kuskokwim River downstream from Bethel (Fig. 2B). The dates of these connections are unknown, but given the low topographic relief in the Yukon-Kuskokwim Delta, when the Yukon and Kuskokwim rivers were connected, gene flow would have been likely among populations on these two rivers.

Historical connections between the Nushagak and the Kuskokwim rivers are also likely. From bathymetric data and known rates of sea level increases following the LGM (Fairbanks 1989), it has been deduced that approximately 11,500 years ago, the lower reaches of these drainages joined near Port Moller and emptied into the Bering Sea at the Bering Canyon on the shelf break (Hopkins 1967; Wilmot et al. 1994; Seeb and Crane 1999; Fig. 2A). In addition, the Mulchatna River (a tributary of the Nushagak) may have connected to the Stony River (a tributary of the Middle Kuskokwim) near Telaquana Lake (Fig. 2C; Maddren 1910). In this study, we used data for western Alaskan chum salmon populations that were genotyped with single nucleotide polymorphism (SNP) and microsatellite markers to ask if contemporary gene flow or historical connections among CSW chum salmon populations allowed gene flow to occur and best explain their present-day spatial pattern of genetic structure.

## Materials and Methods

### Populations and genotype data

Thirty-five populations of chum salmon from western Alaska were genotyped with 12 microsatellites (Kondzela et al. 2013) and 69 SNPs (some nuclear loci and the mitochondrial variants were linked; Garvin and Gharrett 2007; Garvin et al. 2010; Seeb et al. 2011; Tables 1 and 2). Some markers were unavailable for all populations so we used two data sets. The first set included the largest geographic range of populations (*n* = 25) and markers (12 microsatellite, 58 SNP loci; 25P70L). The second set included the largest number of populations within CSW (*n* = 21) genotyped at 50 SNP loci 21P50L. Populations were divided into eight regional groups: A – Kotzebue Sound, B – Norton Sound, C – Lower Yukon, D – Middle Yukon, E – Lower Kuskokwim, F – Middle Kuskokwim, G – Upper Kuskokwim, and H – Bristol Bay (Fig. 2A, Table 1).

**Table 1:** Geographical and run timing information of the samples

Sample location	Code	Regional group	Year	N	Lat	Lon	cLat	cLon	Source	Run timing	25P70L	21P50L
Kelly Lake	Al	Kotzebue sound	1991	96	67.92	−162.35	67.27	−162.48	ADF&G	Summer	Yes	No
Noatak	A2	Kotzebue sound	1991	96	67.98	−162.51	67.49	−161.99	ADF&G	Summer	Yes	No
Kobuk	A3	Kotzebue sound	2000	96	66.92	−160.81	67.06	−156.51	USFWS	Summer	Yes	No
Pilgrim	Bl	Norton sound	2004	96	65.16	−165.22	64.92	−165.10	KWRK	Summer	Yes	No
Snake	B2	Norton sound	2004	96	64.50	−165.41	64.52	−165.41	KWRK	Summer	Yes	No
Unalakleet	B3	Norton sound	2005	96	63.87	−160.79	63.97	−159.94	KWRK	Summer	Yes	No
Black River	CI	Lower yukon	2006	95	62.35	−165.35	62.09	−164.81	ADF&G	Summer	No	Yes
Andreafsky	C2	Lower yukon	1993	93	62.12	−162.81	63.20	−162.58	USFWS	Summer	No	Yes
Achuelinguk	C3	Lower yukon	1989	93	61.96	−162.83	62.01	−162.73	USFWS	Summer	No	Yes
Anvik*	C4	Lower yukon	1989	75	62.68	−160.20	63.12	−160.59	USFWS	Summer	Yes	Yes
Innoko	C5	Lower yukon	1993	86	62.25	−159.56	62.68	−159.56	ADF&G	Summer	No	Yes
Nulato*	C6	Lower Yukon	2003	48	64.71	−158.14	64.65	−158.79	USFWS	Summer	Yes	Yes
Gisasa	C7	Lower yukon	1994	95	65.25	−157.71	64.81	−159.02	ADF&G	Summer	No	Yes
Toklat	Dl	Middle yukon	1994	96	64.45	−150.31	63.82	−150.05	USFWS	Fall	Yes	Yes
Salcha	D2	Middle yukon	1994	96	64.47	−146.98	64.83	−144.71	USFWS	Summer	Yes	No
Goodnews*	El	Lower Kuskokwim	1989	96	59.10	−161.56	59.32	−160.68	USFWS	Summer	Yes	Yes
Kanektok*	E2	Lower Kuskokwim	1989	75	59.75	−161.93	59.84	−160.35	USFWS	Summer	Yes	No
Kwethluk*	E3	Lower Kuskokwim	1989	77	60.81	−161.45	60.20	−159.90	USFWS	Summer	Yes	Yes
Aniak*	Fl	Middle Kuskokwim	1992	95	61.57	−159.49	60.80	−159.51	ADF&G	Summer	Yes	Yes
Salmon	F2	Middle Kuskokwim	2007	96	61.06	−159.20	60.75	−159.69	USFWS	Summer	No	No
Holokuk*	F3	Middle Kuskokwim	2007	63	61.54	−158.59	61.30	−158.31	USFWS	Summer	Yes	Yes
Oskawalik*	F4	Middle Kuskokwim	1994	58	61.75	−158.18	61.56	−157.79	ADF&G	Summer	Yes	Yes
George*	F5	Middle Kuskokwim	2007	96	61.90	−157.71	62.17	−157.26	USFWS	Summer	Yes	Yes
Kogrukluk*	F6	Middle Kuskokwim	2007	96	60.85	−157.85	60.55	−158.29	USFWS	Summer	Yes	Yes
Stony*	F7	Middle Kuskokwim	1994	151	61.77	−156.59	61.16	−154.10	ADF&G	Summer	Yes	Yes
Tatlawiksuk	F8	Middle Kuskokwim	2007	96	61.92	−156.24	62.16	−155.53	USFWS	Summer	No	No
Takotna*	Gl	Upper Kuskokwim	2007	96	62.96	−155.60	62.66	−156.64	USFWS	Summer	Yes	Yes
Big River	G2	Upper Kuskokwim	2008	96	62.61	−155.01	62.69	−154.38	ADF&G	Fall	Yes	No
South fork	G3	Upper Kuskokwim	2008	96	63.09	−154.64	62.06	−153.48	ADF&G	Fall	Yes	No
Meshik	H1	Bristol bay	1989	75	56.81	−158.66	56.60	−158.42	USFWS	Summer	Yes	No
Big Creek	H2	Bristol bay	1988/2000	96	58.29	−157.53	58.18	−155.76	USFWS	Summer	Yes	No
Nushugak*	H3	Bristol bay	1988	75	58.80	−158.63	60.11	−156.99	USFWS	Summer	Yes	Yes
Stuyahok	H4	Bristol bay	1992/1993	87	60.19	−156.29	60.18	−156.15	ADF&G	Summer	No	Yes
Mulchatna	H5	Bristol bay	1994	95	59.95	−156.41	60.61	−154.42	ADF&G	Summer	No	Yes
Togiak	H6	Bristol bay	1993	95	59.08	−160.34	59.19	−160.38	ADF&G	Summer	No	Yes

Lat, latitude; Lon, longitude of each sample location. The cLat and cLon values are the latitude and longitude of the centroid of each drainage. The 25P70L and 21P50L columns indicate whether a population was included in the 25P70L or the 21P50L data set. Populations in gray are the coastal southwestern Alaska (CSW) populations used for the IBD analysis that show the weakest genetic structure.

ADF&G, Alaska Department of Fish and Game; USFWS, U.S. Fish and Wildlife Service; KWRK, Kawerak Inc.,

**Table 2:** ”A”, number of alleles; “*H*_*e*_” and “*H*_*o*_”, expected and observed heterozygosity; “θ”is Weir and Cockerham's *F*_*ST*_, θ_*csw*_ is the *F*_*ST*_ value for CSW populations only

Locus	Locus type	A	*H*_*e*_	*H*_*o*_	θ	θ_*csw*_	D_*EST*_	Source
Okil00	mSAT	23	0.902	0.871	0.012	0.001	0.095	UAF/ABL
Omml070	mSAT	40	0.961	0.955	0.004	0.000	0.099	UAF/ABL
Omy1011	mSAT	30	0.924	0.922	0.009	0.002	0.092	UAF/ABL
One101	mSAT	34	0.896	0.893	0.014	0.001	0.107	UAF/ABL
One102	mSAT	21	0.910	0.886	0.004	0.000	0.041	UAF/ABL
One104	mSAT	30	0.929	0.919	0.018	0.000	0.182	UAF/ABL
One111std	mSAT	93	0.924	0.912	0.016	0.000	0.161	UAF/ABL
One114	mSAT	47	0.922	0.913	0.008	0.002	0.078	UAF/ABL
Ots103	mSAT	43	0.947	0.934	0.008	0.000	0.125	UAF/ABL
Ots3std	mSAT	20	0.767	0.740	0.031	0.003	0.085	UAF/ABL
Otsg68	mSAT	40	0.944	0.925	0.010	0.002	0.138	UAF/ABL
Ssa419	mSAT	21	0.862	0.855	0.012	0.000	0.066	UAF/ABL
AHR178	SNP	2	0.491	0.496	0.011	−0.002	0.011	ADF&G
ARF	SNP	2	0.308	0.289	0.022	0.009	0.010	ADF&G
CCT3220	SNP	2	0.355	0.354	0.009	0.001	0.005	ADF&G
CKS389	SNP	2	0.377	0.363	0.012	0.000	0.006	ADF&G
CL	SNP	2	0.402	0.405	0.005	0.002	0.003	UAF/ABL
COPA	SNP	2	0.057	0.058	0.014	0.005	0.001	ADF&G
ctgf105	SNP	2	0.263	0.266	0.014	0.000	0.004	ADF&G
CTS1627	SNP	2	0.484	0.490	0.003	−0.003	0.003	ADF&G
DM20	SNP	2	0.492	0.508	0.010	−0.002	0.008	ADF&G
EIF4EB	SNP	2	0.082	0.082	0.019	0.006	0.002	ADF&G
ER	SNP	2	0.174	0.166	0.024	0.016	0.005	UAF/ABL
FARSLA242	SNP	2	0.061	0.062	0.030	0.000	0.002	ADF&G
GAPDH	SNP	2	0.488	0.475	0.022	0.000	0.019	ADF&G
GHII	SNP	2	0.406	0.378	0.021	−0.001	0.013	ADF&G
GnRH527	SNP	2	0.358	0.361	0.012	−0.001	0.006	ADF&G
GPH105	SNP	2	0.461	0.425	0.031	−0.002	0.024	ADF&G
hnRNPL239	SNP	2	0.108	0.104	0.017	−0.003	0.002	ADF&G
HP182	SNP	2	0.365	0.350	0.010	−0.002	0.005	ADF&G
HSP90BA299	SNP	2	0.007	0.007	0.000	−0.002	0.000	ADF&G
IGFI1	SNP	2	0.049	0.050	0.018	0.001	0.001	ADF&G
IL8r272	SNP	2	0.183	0.179	0.013	0.000	0.003	ADF&G
IN	SNP	3	0.317	0.300	0.017	0.004	0.008	UAF/ABL
IN1								
IN2								
IS	SNP	4	0.564	0.555	0.017	0.006	0.020	UAF/ABL
ISOII								
ISOP								
KPNA287	SNP	2	0.092	0.089	0.021	0.003	0.002	ADF&G
MAPK1135	SNP	2	0.242	0.245	0.012	0.002	0.004	ADF&G
MARKS	SNP	2	0.426	0.417	0.008	0.010	0.006	ADF&G
MOESIN	SNP	2	0.125	0.124	0.018	0.008	0.003	ADF&G
PER	SNP	2	0.080	0.078	0.029	0.024	0.003	UAF/ABL
PL	SNP	2	0.138	0.138	0.030	0.039	0.005	UAF/ABL
RACP	SNP	2	0.399	0.401	0.012	−0.003	0.007	ADF&G
RAS1	SNP	2	0.422	0.403	0.027	−0.004	0.021	ADF&G
RF	SNP	2	0.450	0.437	0.036	0.004	0.028	ADF&G
RH	SNP	2	0.058	0.057	0.004	0.003	0.000	UAF/ABL
SP	SNP	2	0.499	0.517	0.003	−0.003	0.003	UAF/ABL
TCP178	SNP	2	0.127	0.122	0.020	0.005	0.003	ADF&G
TF278	SNP	2	0.351	0.337	0.054	−0.002	0.027	ADF&G
TSHA1	SNP	2	0.259	0.237	0.012	0.004	0.005	ADF&G
ul519	SNP	2	0.265	0.257	0.020	0.002	0.007	ADF&G
U200	SNP	2	0.486	0.474	0.014	0.000	0.013	ADF&G
U202	SNP	2	0.124	0.122	0.042	0.000	0.005	ADF&G
U212	SNP	2	0.059	0.055	0.028	0.010	0.002	ADF&G
U216	SNP	2	0.245	0.239	0.006	−0.002	0.002	ADF&G
U217	SNP	2	0.488	0.501	0.017	0.002	0.015	ADF&G
U302195	SNP	2	0.412	0.424	0.040	0.012	0.026	ADF&G
U502241	SNP	2	0.212	0.216	0.014	−0.002	0.003	ADF&G
U503272	SNP	2	0.144	0.142	0.003	0.001	0.000	ADF&G
U504	SNP	2	0.498	0.499	0.003	−0.001	0.004	ADF&G
U505112	SNP	2	0.438	0.441	0.007	0.001	0.005	ADF&G
U506110	SNP	2	0.205	0.195	0.028	0.001	0.007	ADF&G
U507286	SNP	2	0.497	0.503	0.006	−0.002	0.006	ADF&G
U509219	SNP	2	0.500	0.510	0.003	−0.001	0.004	ADF&G
U510204	SNP	2	0.304	0.301	0.019	0.006	0.009	ADF&G
U511271	SNP	2	0.156	0.145	0.007	0.002	0.001	ADF&G
U514150	SNP	2	0.251	0.240	0.018	0.001	0.005	ADF&G
VR	SNP	4	0.708	0.683	0.022	0.023	0.050	UAF/ABL
VR1								
VR2								
VR3								
VT	SNP	2	0.472	0.461	0.014	−0.004	0.011	UAF/ABL
ZAN132	SNP	2	0.449	0.451	0.010	−0.003	0.008	ADF&G
MT	SNP	10	0.250	N/A	0.033	0.020	N/A	UAF/ABL
MT5	SNP							UAF/ABL
MT12	SNP							UAF/ABL
MT18	SNP							UAF/ABL
MT21	SNP							UAF/ABL
MT27	SNP							UAF/ABL
CR30	SNP							Sato et al. 2004;
CR231	SNP							Sato et al. 2004;
CR386	SNP							Sato et al. 2004
Overall	mSAT	36.8	0.907	0.894	0.012	0.001	0.090	
Overall	SNP	2.2	0.305	0.320	0.016	0.001	0.003	
Overall	SNP & mSat	8.2	0.408	0.481	0.015	0.001	0.009	

*ϕ*_*ST*_, for the mitochondrial locus. Grey boxes indicate outlier loci. Some SNP loci are composed of multiple linked SNPs (indented). *D*_*EST*_ is Jost's D. “Source” indicates the agency that provided the tissue or generated the original data for that marker.

UAF, University of Alaska Fairbanks; ABL, Auke Bay Labs.

### Hierarchical *G*-test

Genetic divergence within and among regional groups of chum salmon populations was tested with log likelihood ratios (*G*-tests). *G*-tests can be summed across loci and different regional groups to identify significance at hierarchical levels. Simulations have shown that *G*-tests can have high power but high type I error (Ryman et al. 2006) because they do not approximate a chi-square distribution for loci with low numbers of alleles. However, the test performs well when there are more than four alleles at a locus. Therefore, for the multi-allelic microsatellite loci, if the expected number of alleles at each locus for the smallest sample was less than four, we combined that allele with the next largest sized allele at that locus for all populations. A *G*-statistic was calculated for each locus and significance was determined by summing over all loci for each regional group. Regional groups were based on the geographical locations from which the samples were taken and on the present-day courses of the river systems (Fig. 2). An approximate *F*-test was constructed to compare the extent of divergence among and within populations (Gharrett et al. 1987):


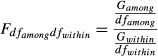


where *df,* the degrees of freedom.

### Measures of divergence

Allele numbers, expected and observed heterozygosities,*θ* for nuclear loci (analogous to *F*_*ST*_; Weir and Cockerham 1984) and Φ_*ST*_ for the mitochondrial variants (Excoffier et al. 1992) were estimated with the software package Genetic Data Analysis (GDA) (Lewis and Zaykin 2001). Jost's *D*_*EST*_ was calculated with the function “D_Jost” in the “adegenet” package (Jombart 2008) in the R environment and *θ* was calculated for each locus with data from only the CSW populations (θ_*CSW*_). *F*_*SC*_ and *F*_*CT*_ were estimated with Arlequin 3.5 (Excoffier and Lischer 2010).

### Principal components analysis

A principal components analysis (PCA) was performed on the allele frequency data from the 25P70L data; all allele frequencies were arcsine-square root transformed prior to the analysis in SYSTAT (Sokal and Rohlf 1994). Microsatellite alleles were binned as described for the *G*-test.

### Neighbor-joining tree

A neighbor-joining tree was constructed from observed allele frequency data with Cavalli-Sforza and Edwards (1967) chord distances that were calculated with the 25P70L data. A consensus tree was produced from 1000 neighbor joining trees generated by bootstrapping loci and then combining them with the CONSENSUS program in the PHYLIP package (Felsensein 2004). All nodes with bootstrap values greater than 0.5 were labeled on the majority-rule tree.

### Outlier analysis

Genetic markers that showed larger than expected *F*_*ST*_ values as compared to a null distribution based on expected heterozygosity were identified with Arlequin 3.5 (Excoffier and Lischer 2010). This method takes into account hierarchical structures of populations, which is common for salmon. We used the 25P70L data for this analysis, but removed data from some populations.

The Salmon and Tatlawiksuk rivers were excluded because data were unavailable for all 70 loci, and fall-run Upper Kuskokwim fish and Middle Yukon fish were excluded because they have different spawning times than summer-run fish (Gilk et al. 2009) and gene flow is unlikely. The samples from the Takotna River from the Upper Kuskokwim drainage were included in the analysis because they are summer-run fish and they clustered with the samples from the Middle Kuskokwim River. We tested for outliers with data from (1) only SNPs, (2) only microsatellites, and (3) both marker types combined.

### Isolation by distance

Prior to the IBD analysis, we eliminated loci potentially under positive directional selection and tested for IBD among and within summer-run chum populations from the Yukon, Kuskokwim, and Nushagak drainages as they exist today and again assuming historical connections among those systems with the program GENEPOP (Rousset 2008). The IBD between the Yukon and Kuskokwim drainages was performed the 25P70L data. Only one population from the Nushagak drainage was available for analysis within the 25P70L data set, but samples from more populations were available for the 21P50L data set (Table 1), so we used allele frequencies from the Nushagak and Kuskokwim rivers in the latter data set to test for IBD between those drainages. In order to determine if the exclusion of 20 loci affected the IBD results for the test between the Kuskokwim and the Nushagak rivers, we tested for IBD between the Yukon and Kuskokwim drainages with the 21P50L data to see if the results were similar to the analysis with the 25P70L data. The data from the Norton Sound, middle Kuskokwim, or fall-run upper Kuskokwim populations were not used for any of the IBD analyses; our interest here is only the CSW populations.

The relationship between the genetic and water distances (*d*) is estimated from:


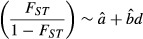


where â is the intercept and the slope, 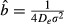, is inversely proportional to the effective linear density of individuals (*D*_*e*_) and the mean-squared parent-offspring distance (σ^2^ GENEPOP (Rousset 1997). The parent-offspring distance can be considered a measure of physical dispersal (straying). Significance was determined with a Spearman rank correlation coefficient. We analyzed the same data with the Mantel test available with the “ade” package in the R environment to determine the correlation coefficient because GENEPOP does not provide one.

The present-day great circle geographic distances were drawn between the sample location of each population through freshwater or marine environments with Google Earth™. The latitude and longitude coordinates identify the weir locations or sonar stations where the samples were taken, which are not necessarily the locations of the spawning populations. Therefore, we used the latitude and longitude coordinate for the centroid of each drainage provided by the sub-watershed delineation tool in the Riverscape Analysis Project (Whited et al. 2012). The distances measured among populations were not significantly altered whether the centroid or weir locations were used, but the centroid provided a means to consistently measure distances among populations.

The possible historical connections between the Yukon and Kuskokwim rivers and between the Kuskokwim and Nushagak rivers were drawn in Google Earth™ by following published reconstructed connections (Maddren 1910; Shepard and Wanless 1971). We assumed that the present-day populations were located in the past where they are today.

### Dispersal distance

The IBD analysis provides an estimate for the slope 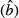 of the regression of the genetic distances on the geographic distances. Rearrangement of the equation give the relationship 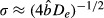. The approximate 95% confidence interval of the geographic distance of parent-offspring pairs is 4 *σ* for a normal distribution, which provides an estimate of the dispersal distances of individuals within populations (e.g., Gharrett et al. 2012).

We calculated density with empirical data from the Kuskokwim River and the slope determined in GENEPOP with the 25P70L data. We used estimates from Bue et al. 2007 for the annual census size (*N*_*c*_) of the Kuskokwim River. Although this is likely an underestimate, it provides an upwardly biased estimate of dispersal distance and is therefore a conservative over-estimate of the potential gene flow among populations. The annual *N*_*c*_ estimate also includes fall-run fish, but those populations are small relative to the summer-run populations and the estimate of Bue et al. (2007) is conservative. The density 
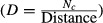
 was estimated by dividing the harmonic mean of the annual census size estimates from Bue et al. (2007) by the total linear distance of the Kuskokwim River, which was calculated by summing the linear distances between the centroids of all of the major drainages for summer-run chum salmon (Table 1).

The effective density 
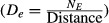
 was determined from a sensitivity analysis of plausible ratios of 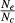 after accounting for two factors: First, chum salmon have overlapping generations and an average age of return of 4 years in the Northern Hemisphere (Salo 1991), which means that the annual *N*_*c*_ is a portion of the existing population. Therefore, we multiplied the *N*_*c*_ by four to account for potential future breeders that remained at sea. Second, the effective population size (*N*_*e*_) can be considered to be the efficiency of passing genes from one generation to the next. For salmon, the determination of *N*_*e*_ can be challenging because of fluctuating population sizes and overlapping generations (Falconer and Mackay 1996; Waples 2002). Although our methods made some large assumptions, our purpose was to estimate the order of magnitude of the dispersal distance (i.e., tens, hundreds, or thousands of kilometers). Therefore, we used a range of 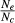 ratios from 0.0125 to 0.75, which brackets previous estimates of *N*_*e*_ for populations of chum salmon in Norton Sound (Burkhart and Dunmall 2005; Olsen et al. 2006) and reported the mean dispersal distances for the range of 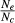 ratios.

## Results

### Hierarchical *G*-test and divergence

The hierarchical *G*-tests with the data from the 25P70L data set were all significant (*P* < 0.001) except for the populations from the Middle Kuskokwim River (Table 3), which suggests structure exists within and among regions. However, the total divergence among populations within regions was less than the total divergence among regions (*F* = 2.53, *P* < 10^−6^), which may reflect the weak genetic structure of the CSW populations. The tests with the 21P50L data set were similar (Table 4). Finally, the θ_*CSW*_ values over all loci for CSW populations were an order of magnitude lower compared to the estimates for all of the populations in western Alaska (Table 2).

**Table 3:** Log-likelihood ratio (*G*) tests for the 25P70L data set

Regional group	*G*	df	*P*-value
Kotzebue	1358.5	358	<10^−6^
Norton sound	657.6	358	<10^−6^
Lower yukon	239.9	179	1.3 × 10^−3^
Middle yukon	437.2	179	<10^−6^
Upper KUSKOKWIM	1349.0	358	<10^−6^
Middle Kuskokwim	391.8	358	1.1 × 10^−1^
Lower Kuskokwim	1030.5	895	1.1 × 10^−3^
Bristol bay	969.6	358	<10^−6^
Total within	6434.1	3043	<10^−6^
Total among	6694.3	1253	<10^−6^
Total	13128.4	4296	<10^−6^

**Table 4:** Log-likelihood ratio (*G*) tests for the 21P50L data set

Regional Group	*G*	df	*P*-value
Yukon	414.35	300	1.3 × 10^−5^
Kuskokwim	660.89	450	<10^−5^
Bristol Bay	527.27	150	<10^−5^
Overall	1602.51	900	<10^−5^

### Principal components analysis

**Figure 3:**
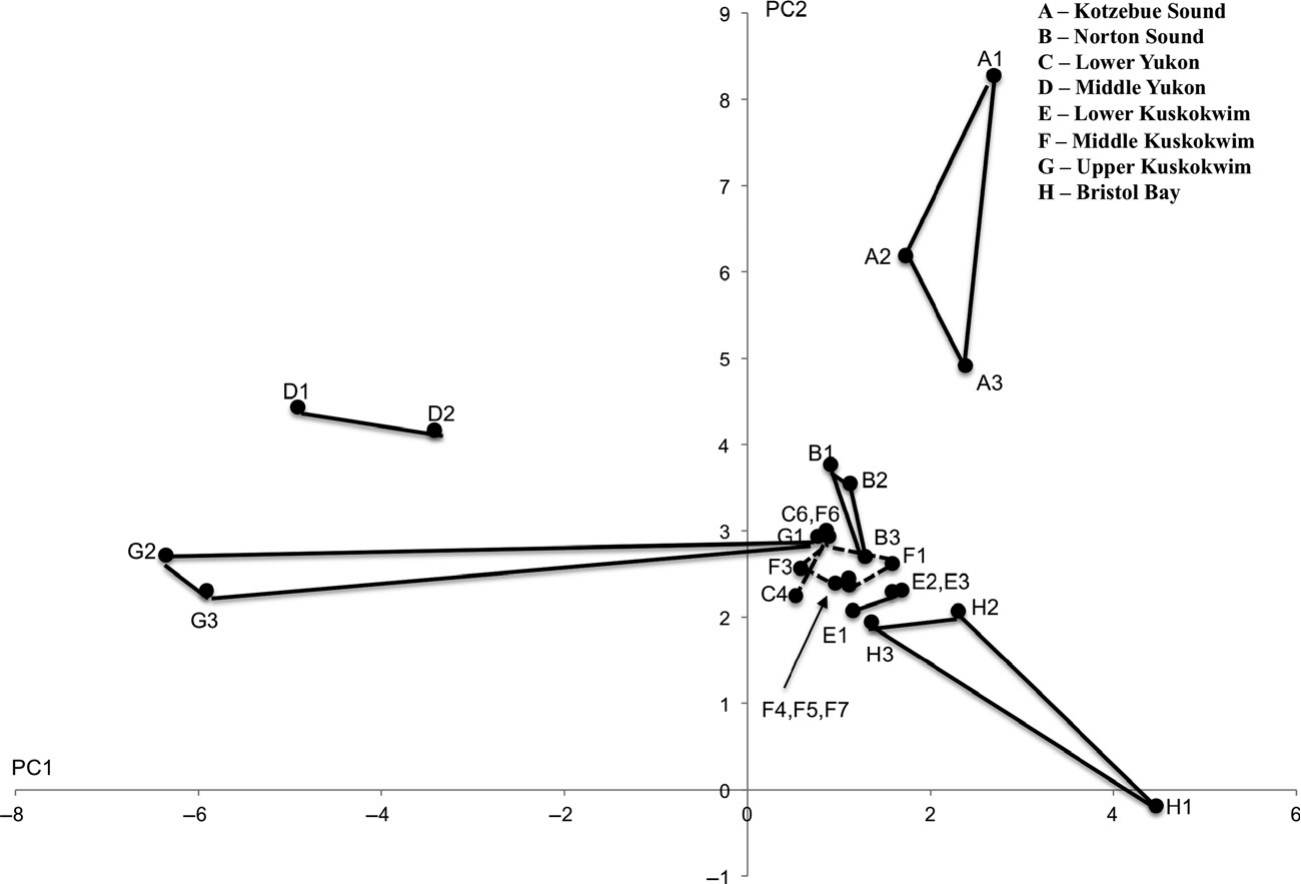
Principle components analysis. The alpha-numeric symbols correspond to Table 1. The first and second components show distinct clusters for Kotzebue Sound, Middle Yukon River, northern Norton Sound, southern Bristol Bay, and fall-run Upper Kuskokwim River populations.

The PCA indicated little divergence among southern Norton Sound, Lower Yukon, Kuskokwim and Northern Bristol Bay populations (Fig. 3). The first two components explained 31.9% of the variance and the third and fourth components added 9.23% and 5.94% of the variance respectively. The Kotzebue Sound samples (A1–A3), the Middle Yukon samples (D1, D2), and the fall-run Upper Kuskokwim populations (G2, G3) were clearly divergent from all others. The samples from the Nushagak River (H3) in eastern Bristol Bay, the Takotna River (G1) from the upper Kuskokwim system, the Unalakleet River (B3) in southern Norton Sound, the Lower Yukon (C4, C6), Lower Kuskokwim (E1–E3), and Middle Kuskokwim samples (F1, F3–F7) formed one indistinguishable cluster. The samples from southern Bristol Bay (H1, H2), and northern Norton Sound populations (B1, B2) demonstrated visible separation from this central group.

### Neighbor-joining trees

**Figure 4:**
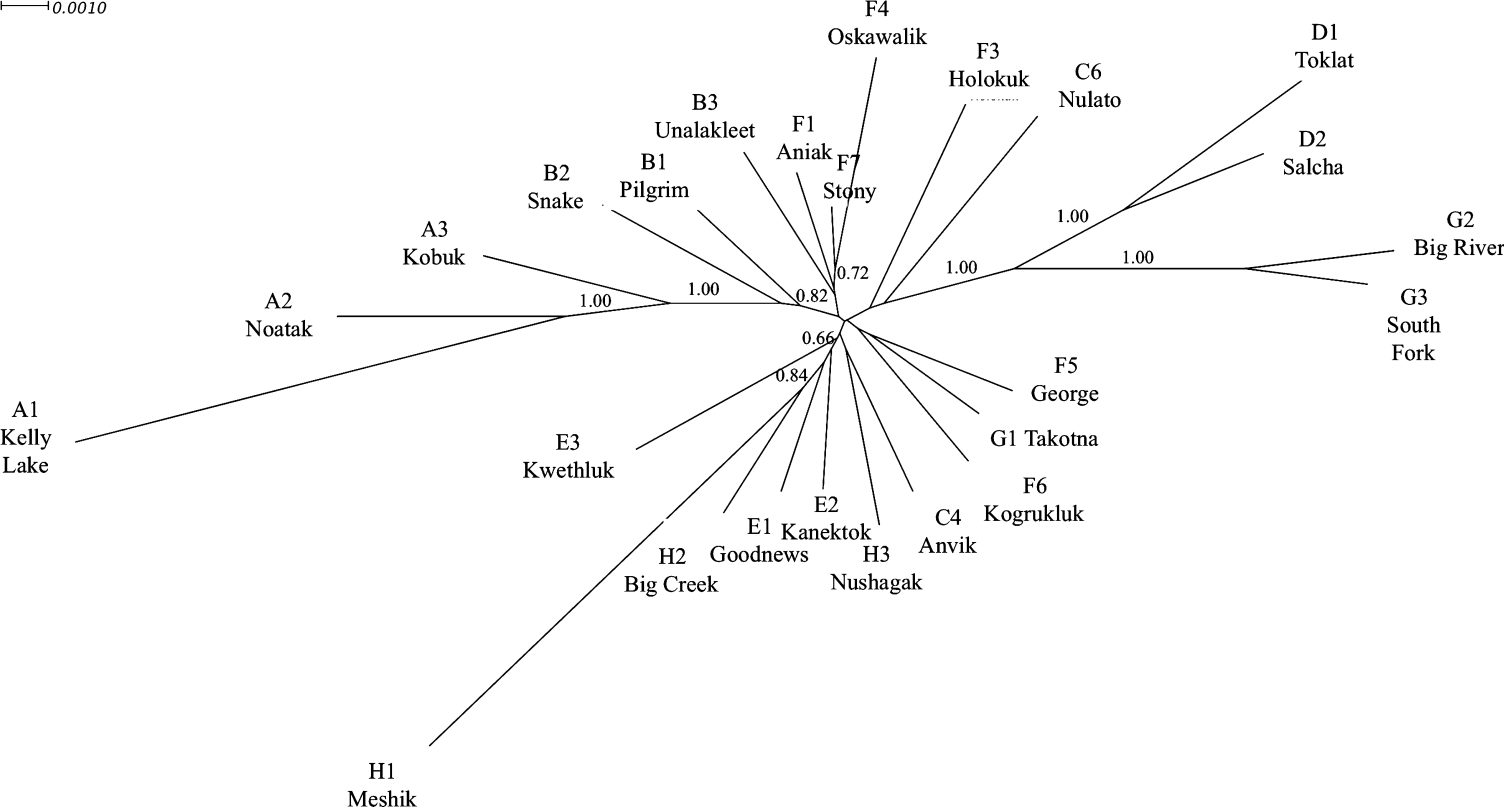
Majority-rule neighbor-joining tree from the 25P70L data set. The edge lengths reflect genetic distance and alpha-numeric codes correspond to populations in Table 1 and Figure 1. Numbers indicate bootstrap support from the consensus tree.

We included bootstrap values from the consensus tree to create a majority rule neighbor-joining tree, which supported the PCA (Fig. 4). The Kotzebue Sound, Middle Yukon, and Upper Kuskokwim populations have bootstrap estimates of 1.0 for the nodes connecting them and the bootstrap estimates for the northern Norton Sound and Eastern Bristol Bay populations are relatively high (greater than 0.8); but the tree reveals weak genetic structure among CSW populations (bootstrap values are all less than 0.55).

### Outlier analysis

**Figure 5:**
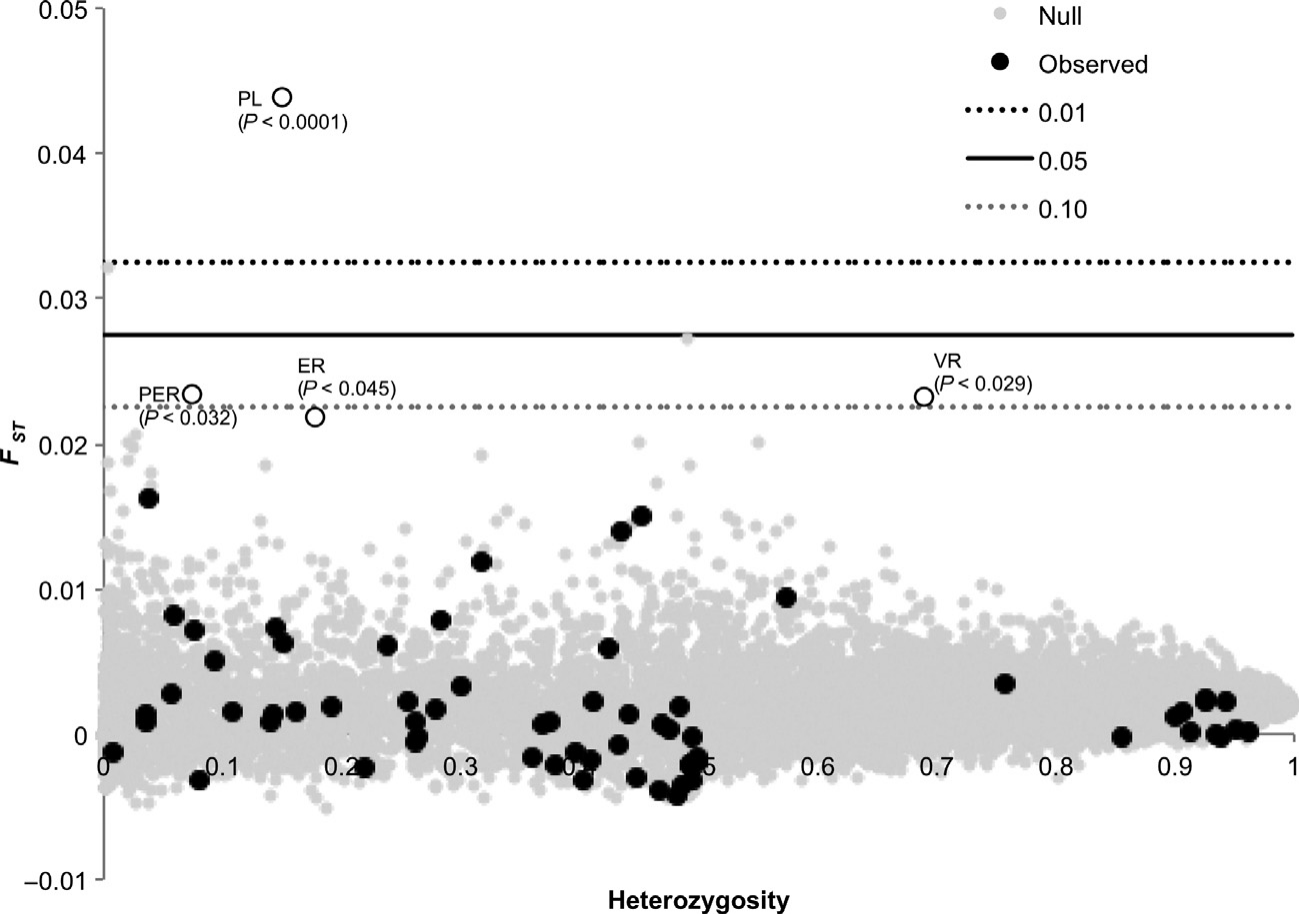
Outlier analysis from 50 nuclear single nucleotide polymorphism (SNPs) and 12 microsatellites among the coastal southwestern Alaska (CSW) populations. The gray symbols denote the simulated null distribution based on a hierarchical population structure. The black symbols are the values for the empirical data. Outlier loci are indicated with empty circles, and the name from Table 2 and associated *P*-values (*P*) are shown next to each circle.

The outlier analysis identified four loci that differed significantly (*P* < 0.05) from the null distribution and indicated that those or linked loci did not conform to neutral expectations (Fig. 5). The same four SNPs were identified as outliers when only the SNP data were used and when the combined SNP and microsatellite data were used. We removed the data for these four loci prior to the IBD analysis so that we could satisfy the assumption of neutrality.

### Isolation by distance

**Figure 6:**
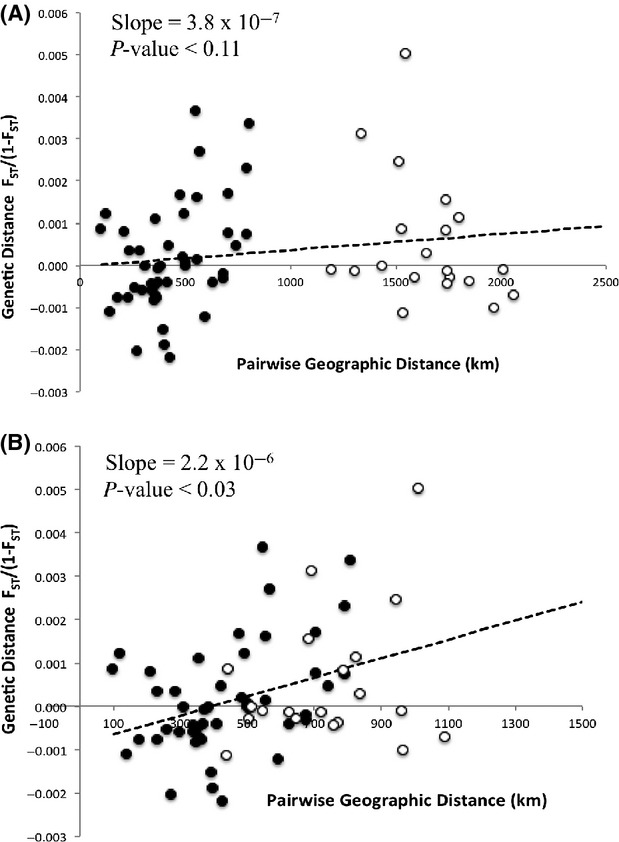
(A) Isolation-by-distance (IBD) analysis between the Yukon and Kuskokwim rivers with present geographical distances with data from the 25P70L data set. The slope of the line and the *P*-value are given. Open circles are pairwise comparisons between drainages, filled circles are pairwise comparisons within drainages. (B) Isolation by distance between the Yukon and Kuskokwim rivers based on historical connections. The same genetic data were used as in 5a. The slope of the line and the *P*-value are given. Open circles are pairwise comparisons between drainages, filled circles are pairwise comparisons within drainages.

The *Kuskokwim – Yukon* log-likelihood ratio tests, the PCA, and the neighbor-joining tree confirm earlier published results (Wilmot et al. 1994; Seeb and Crane 1999; Seeb et al. 2011) that demonstrate weak genetic structure among CSW populations. If the weak structure resulted from contemporary gene flow, one would expect there to be no IBD signal within or among drainages. The test of the slope for IBD with data from 66 loci (the 25P70L data minus the outliers) and pairwise geographic distances as they exist today among the populations from the Lower Yukon and Kuskokwim rivers did not differ significantly from zero (*P* < 0.11). In contrast, the test for IBD was significant (*P* < 0.03) when the geographic distances were calculated assuming an historic connection between those rivers and the slope of the line was approximately fivefold higher (Fig. 6). The tests for IBD with data from the 21P50L data set were similar when compared to the values when 66 loci were used (data not shown). This suggests that 50 loci provide sufficient information for the IBD analyses, which allowed us to include more populations in subsequent analyses.

**Figure 7:**
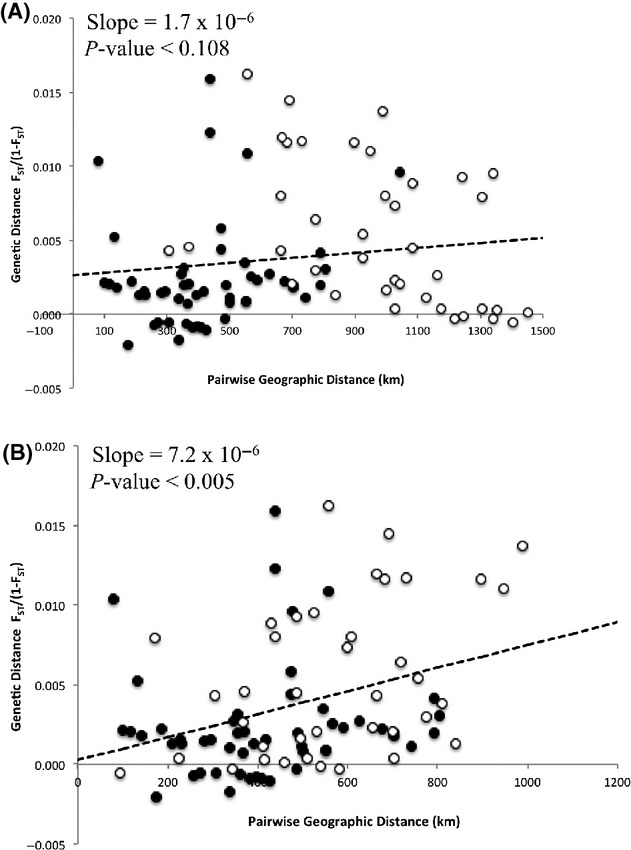
(A) Isolation by distance between the Kuskokwim and Nushagak rivers with present geographical distances. Genetic data for the Kuskokwim and Nushagak populations were taken from the 21P50L data set. The slope of the line and *P*-value given. Open circles are pairwise comparisons between drainages, filled circles are pairwise comparisons within drainages. (B) Isolation by distance between the Kuskokwim and Nushagak based on historical connections. The same genetic data were used as in 6a. The slope of the line and the *P*-value are given. Open circles are pairwise comparisons between drainages, filled circles are pairwise comparisons within drainages.

The *Kuskokwim – Nushagak* IBD was not significant if present-day connections were assumed among populations but the test was significant (*P* < 0.005) when the connection between the Stony and Mulchatna rivers was assumed (Fig. 7). We also tested for IBD within the Yukon and Kuskokwim rivers assuming present day drainage patterns to determine if the slopes of the lines were similar, which might suggest parallel historical demographic processes. The slope of the test for IBD for the seven populations within the Yukon did not differ significantly from zero (*P* < 0.461), but the slope for the test for IBD within the Kuskokwim was significant (*P* < 0.04). The IBD analysis within the Kuskokwim was similar for both data sets (results not shown). There were insufficient samples to test for IBD within the Nushagak River for either data set.

### Dispersal distance on the Kuskokwim River

If long distance straying among CSW populations is responsible for the weak genetic structure, then estimates of dispersal should be large. We used the slope and the mean density from the IBD analysis with data from the Kuskokwim River populations in the 25P70L data set (SNP and microsatellite putatively neutral markers) to estimate the straying distance for 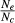 ratios that ranged from 0.0125 to 0.75 (Table 5). The straying distances extended from 28.7 to 222.3 km. When the slope for only the SNP markers was used, the estimates ranged from 11.1 to 85.8 km.

**Table 5:** Dispersal distances estimated with the slope of the IBD analysis from the Kuskokwim River, 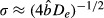

*N*_*E*_/*N*_*C*_ ratio	0.013	0.025	0.050	0.100	0.250	0.500	0.75
*N*_*E*_	57,000	115,000	230,000	460,000	1150,000	2300,000	3450,000
*D*_*E*_ (fish/km)	29.2	58.4	116.9	233.7	584.3	1168.6	1752.9
Dispersal (km)	222.3	157.2	111.2	78.6	49.7	35.2	28.7

## Discussion

The IBD analyses support our hypothesis that the reduced genetic divergence among CSW chum salmon populations is a result of past physical connections among now divided drainages, not from contemporary migration among populations. Erosion of genetic divergence from straying could result either from high levels of long-distance migration or serial migration among neighboring populations, but the dispersal distances do not approach those necessary for long distance migrations; and they are likely over-estimates because we used underestimates of the total census size. Multiple serial migrations among neighboring populations are unlikely given that there is a signal for IBD within the Kuskokwim River, but not between the Yukon and Kuskokwim or between the Kuskokwim and Nushagak drainages and few populations exist between the Yukon and Kuskokwim river mouths to act as vectors for dispersal. A more detailed historical exploration of when and how these connections may have occurred could provide useful information for predicting the response of the present day populations to climate change or reveal subtle genetic structure that may not be evident if groupings are based on present-day geographical and fluvial information.

Chum salmon populations between Japan and Kamchatka and between the Gulf of Alaska and the Pacific Northwest have genetic diversity estimates that are several-fold higher than for populations in the geographic area between Kotzebue Sound and Bristol Bay (Seeb and Crane 1999; Beacham et al. 2009b; Seeb et al. 2011). The exceptions are populations of fall-run fish that spawn in the Yukon and Kuskokwim rivers, which were not the focus of this study. The genetic divergence of the CSW populations nested within western Alaska is further reduced, which could be explained by two processes that occurred during the LGM and from the Pleistocene/Holocene transition to the present; (1) extirpation and (2) dispersal into a dynamic ecosystem in which major river systems were repeatedly altered. Historical evidence indicates at least two significant extirpations and several instances of major modifications to river drainages that occurred during this period.

The first population reduction likely occurred during the LGM. Archaeological evidence from Broken Mammoth in central Alaska near the Tanana River revealed that one or more species of salmonid were present in central Alaska approximately 14,000 years ago (Hoffecker and Elias 2003), and certainly freshwater species survived the LGM given the likely suitable habitats within the fluvial systems of the Bering shelf (McPhail and Lindsey 1986). But paleo-climatic data suggest that there were extended periods of unfavorable conditions for anadromous salmon (Sancetta 1983; Mann and Hamilton 1995). The rivers as they existed at the LGM would have entered the Bering Sea at its present-day shelf break and estuarine habitat that is necessary for the early life history of chum salmon may have been greatly reduced (although not necessarily absent). In addition, the climatic conditions likely caused the Bering Sea to be ice-covered for 9 months of the year, and diatom microfossil assemblages from sediment cores suggest the Bering Sea during the LGM was much less productive than it is today.

Populations that may have survived the LGM would resemble those in similar environmental conditions today (e.g., the Beaufort and northern Chukchi seas); the populations were probably small and ephemeral. However, as sea levels rose, the shorelines retreated rapidly (Fairbanks 1989; Manely 2002) and created potential estuarine habitat on the relatively flat newly formed coastline. By about 12,000 years ago, the connection between the Bering and Chukchi seas had been re-established (Keigwin et al. 2006) and the planktonic productivity in the Bering Sea began to increase (Sancetta 1983; Mann and Hamilton 1995). Beringian chum salmon spawning habitat flooded by rising seas after the LGM would have forced surviving populations to colonize new habitat.

It is likely that the CSW summer-run chum salmon are either the descendants of a paleo-Beringian invasion from the rising seas, expansions from small populations that survived the LGM, or both. By approximately 5000 years ago, the Bering Sea had reached its current level, the present shorelines had been established (Manely 2002) and the Yukon River mouth moved north to flow into Norton Sound (Nelson and Creager 1977; but see Dupŕe 1988; Shaw 1998). Presently, about one-third of the flow of the Yukon River is due to glacier meltwater (Brabets et al. 2000); any substantial retreat of glaciers would have caused changes in the flows of the Yukon River and its drainages. In addition, precipitation in interior eastern Beringia from the LGM to the mid-Holocene was lower than present (Barber and Finney 2000), which likely would have also reduced river flows. Populations in the lower Yukon and Kuskokwim rivers would likely have formed a large meta-population that was repeatedly connected, disconnected, and reconnected as the Yukon River meandered across the broad flat plain of Beringia in response to sea level rise and variable discharges from glaciers that melted with the warming climate.

The weak genetic structure that is present today may indicate that these large meta-populations have not yet reached migration-drift equilibrium. If chum salmon have been present in these drainages since the Holocene Thermal Maximum (roughly 1200 generations) there should have been time to establish equilibrium (Waples et al. 2008), but this state is reached at a rate that is proportional to the effective population size and inversely proportional to the migration rate in a population ((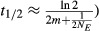; Crow and Aoki 1984). Low gene flow or large effective population size would retard progress toward equilibrium.

Previous work demonstrated an IBD pattern among Yukon populations (Olsen et al. 2008), but we did not. Our results are consistent with the idea that as the mouth of the Yukon River shifted to the north, it may have disrupted population structure in that habitat and erased IBD patterns that had been previously established. The analysis of Olsen et al. 2008 included populations from the Koyukuk and Tanana drainages further upstream from our analysis as well as an isolated divergent population (California River). Future work with more populations from the lower Yukon drainages may reveal the discrepancy between our results and those of Olsen et al. 2008.

Subsequent to the Holocene Thermal Maximum, several periods of glacial advance that occurred within the Neoglacial Period (Calkin et al. 2001) were followed by rapid warming events that may have altered discharge rates and established migration corridors. Paleoclimatic data from Ongoke Lake in southwestern Alaska indicate that approximately 1600 years ago this region experienced the coldest, driest climate in the past 2000 years (Chipman et al. 2008) (Figure 2a), known as the First Millenial Cold (FMC) period. Other work has established that glaciers advanced during this same period, which was followed by a warmer climate and glacial retreat (Wiles et al. 2008; Barclay et al. 2009). Three distinct periods of glacial advance occurred during the Little Ice Age (ca. 1250–1900 before present; Mann 2002; Wiles et al. 2004). The cooler climate caused the Kaskawulsh Glacier at the southern end of Kluane Lake in the Yukon Territory to advance (Fig. 2A) and block the outlet of the lake (Clague et al. 2006). The rise of the lake level caused the outlet to form at the lake's southern end and empty into the White River, which now contributes 10% to the flow of the Yukon River (Brabets et al. 2000). Finally, glaciers in the Ahklun Mountains in southwestern Alaska that formed during the Little Ice Age were reduced by 50% in volume during the subsequent warm period (Levy et al. 2004). Any or perhaps all of these events may have disrupted IBD patterns that had formed in CSW populations after they had expanded from the LGM.

It is also possible that reduced genetic divergence among CSW populations was caused by local extirpations followed by dispersal from a single source. Severe reductions of salmon runs in large areas of Alaska are suggested by historical abundances of sockeye from Kodiak Island (Finney et al. 2002), and archaeological evidence from native communities near Cape Nome in Norton Sound (so-called Norton Phase) that showed those communities ceased to use salmon as a food resource for nearly 300 years even though they had done so for the 1500 years prior to that period (Bockstoce 1973, 1979; Yesner 1998). The cold and dry conditions of the FMC may have created unsuitable habitat in the topographically flat areas of CSW and salmon populations may have declined substantially between 1600 and 1200 years ago. Reduced water temperatures may also have limited the amount of viable chum salmon habitat. The development of salmon eggs is tightly coupled to freshwater temperature so that fry emerge when food resources are available for growth (Beacham and Murray 1990), and chum salmon depend on oxygenated water from either turbulent areas of the river or upwelling from groundwater (Groot and Margolis 1991).

This possible population reduction could also explain the genetic similarity of the populations from the Lower Yukon, the Kuskokwim, and the Nushagak rivers to southern Norton Sound drainages, which do not demonstrate historical connections to the Yukon River; they could have been recolonized at the same time as Yukon River populations. Future geological or hydrological work focused on the timing of the most recent connections between the Yukon and Kuskokwim rivers and the Kuskokwim and Nushagak rivers may provide support for one of these hypotheses as could an analysis of past salmon abundance among these populations that is similar to the one conducted by Finney et al. (2002) on Kodiak Island.

A more recent timing for extirpation, recolonization and/or geneflow is suggested by the fact that the divergence estimates of CSW populations are an order of magnitude lower than for chum salmon in western Alaska overall (θ_*CSW*_ = 0.001 versus θ = 0.016) and recent colonizations by populations of other species of salmon display much greater divergence. For example, the observed divergence estimates among Chinook salmon that were introduced into New Zealand rivers at the end of the 19th century (Kinnison et al. 2002) and pink salmon that colonized new habitat as glaciers receded in Glacier Bay National Park about 125 years ago (Kondzela 2010) are an order of magnitude higher than the CSW populations. Chinook salmon would be expected to show greater divergence and the anthropogenic introduction produced a colonization mechanism that was likely very different that western Alaskan chum salmon, but pink salmon demonstrate IBD patters similar to those of chum salmon (Quinn 2005) and would have followed a natural progression of colonization.

Regardless, the difficulties associated with applied genetic work to chum salmon populations from these dynamic coastal regions of western Alaska may be the result of very recent events coupled with a long-term history of environmental disruptions and drainage interconnections. This conclusion combined with the fact that large numbers of neutral microsatellite, allozyme, and SNP markers have been unable to demonstrate more divergence than we have shown here suggests that the addition of more neutral markers may be inadequate to demonstrate significant divergence among CSW populations, which are necessary for applied genetics work. As more data sets become available, they can be merged to further test the hypotheses here. Markers responsible for local adaptation may demonstrate larger divergence estimates than our current analysis.
